# Supplementary material for: Effects of Embryonic Thermal Manipulation on Body Performance and Cecum Microbiome in Broiler Chickens Following a Post-Hatch Lipopolysaccharide Challenge
Source: Animals (Basel). 2025 Apr 17;15(8):1149. doi: 10.3390/ani15081149 (PMC12024374; doi:10.3390/ani15081149)
Supplement: Supplementary file 1 [file animals-15-01149-s001.zip › animals-3559828-supplementary.pdf]

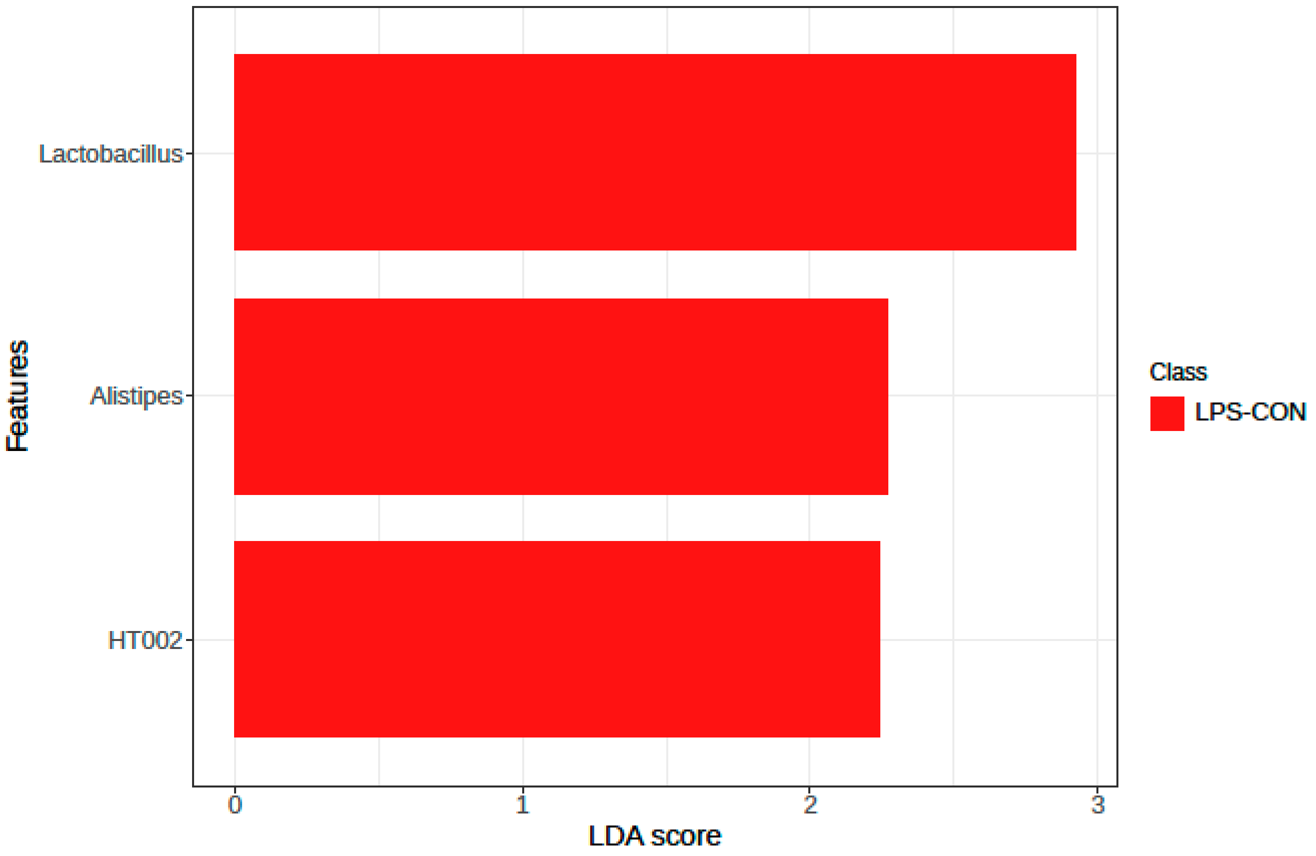

Figure S1. Represent Linear Discriminant Analysis (LDA) score plot at genus level. Support the hypothesis that LPS injection increases lactic acid produces bacteria. However, when applied The Kruskal-Wallis H-test for the relative abundance comparison by STAMP no significant differences were observed between groups.

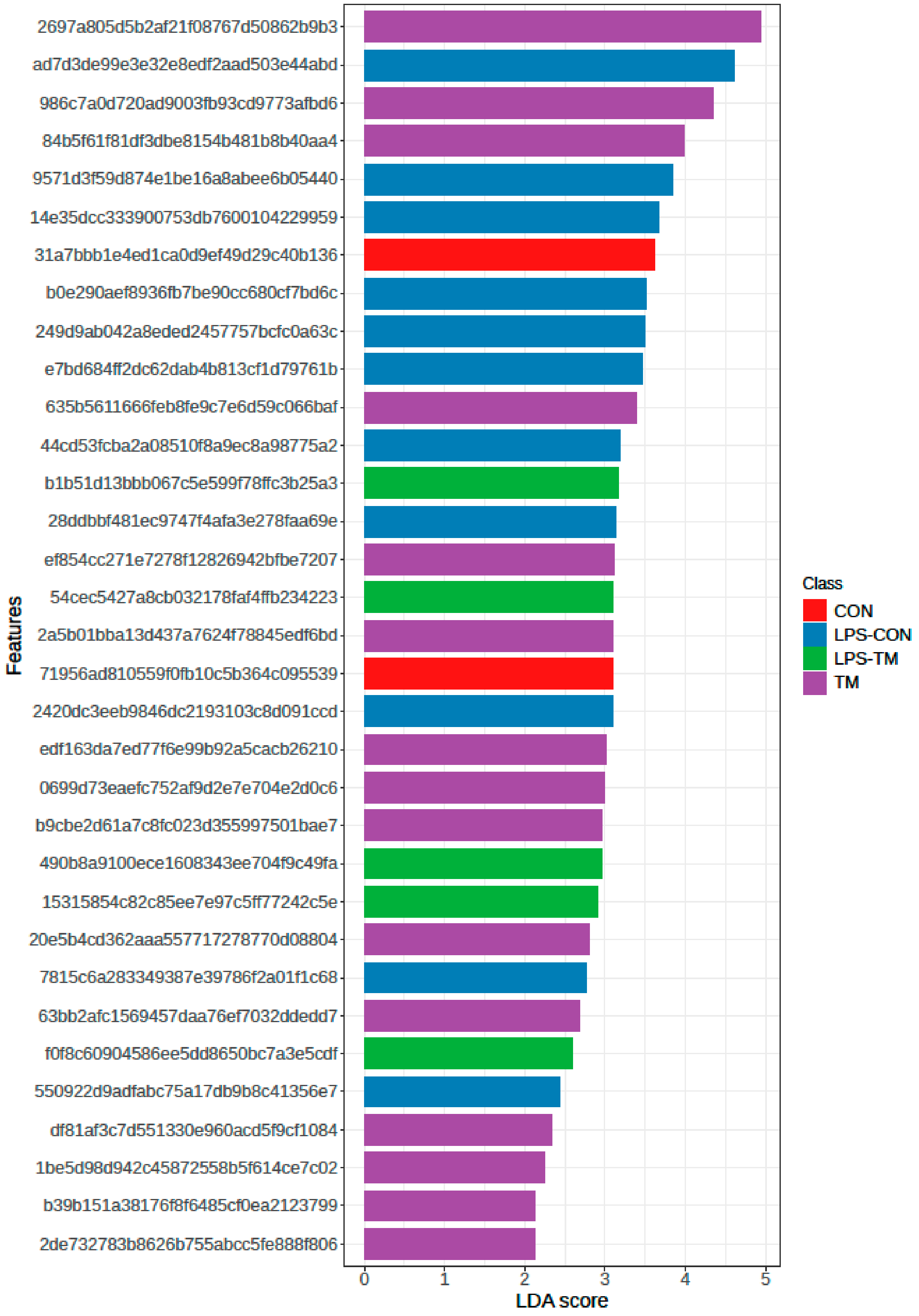

Figure S2. Represent Linear Discriminant Analysis (LDA) score plot at feature level. Highlighting the distinctive bacterial taxa associated with each group. which support the hypothesis that thermal manipulation during embryogenesis modulate post-hatch gut microbiome.

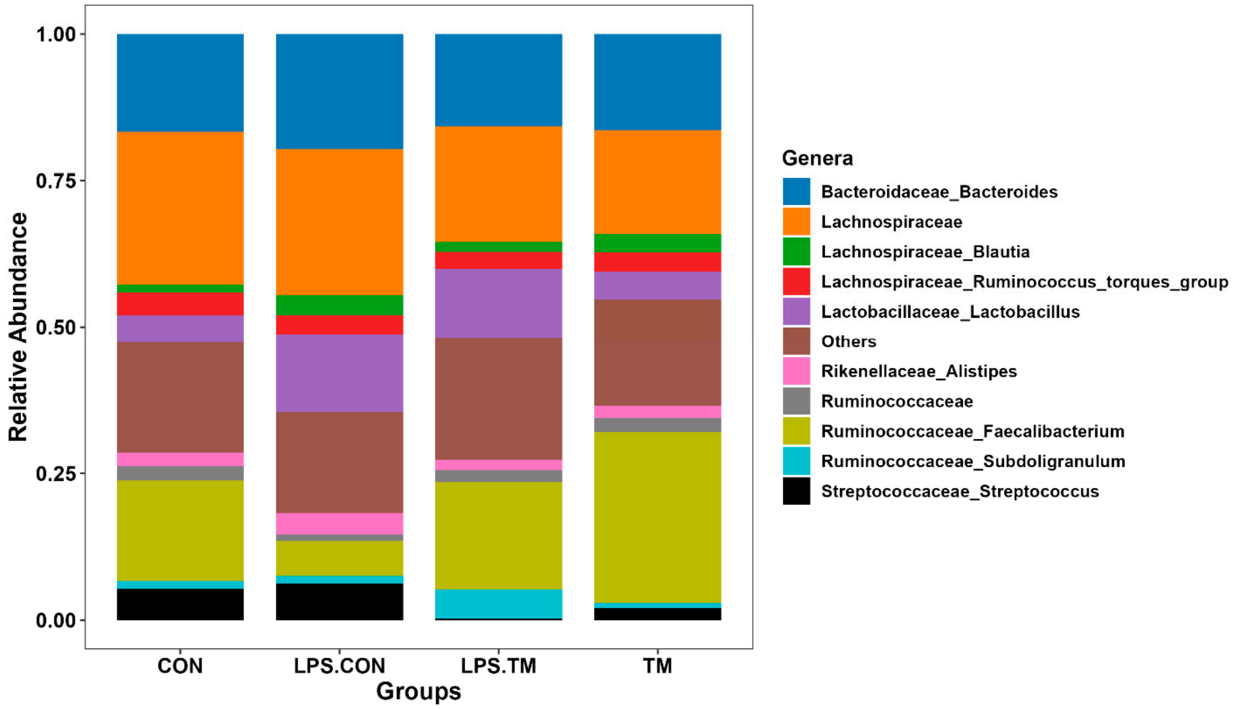

Figure S3. Cecum microbiota composition at the genus level across experimental groups. The LPS-CON group exhibited a lower relative abundance of *Ruminococcaceae_Faecalibacterium* and a higher relative abundance of *Lactobacillaceae_Lactobacillus* compared to other groups. Additionally, *Streptococcaceae_Streptococcus* was more abundant in control-related groups.

QIIME 2 2024.8 version was used for microbiome bioinformatics [1]. ASV-based analysis was applied to the 16S rRNA raw sequence data by the QIIME 2- DADA2 plugin, which provides raw sequence data quality filtering and denoising [2]. A phylogeny was constructed using FastTree 2 (via q2-phylogeny) after all ASVs were aligned with MAFFT Multiple Sequence Alignment Software Version 7 (via q2-alignment) [3,4]. q2-feature-classifier, which uses the Naive Bayes Silva 138 99% OTUs full-length sequences taxonomy classifier, was used to assign taxonomy to ASVs [5,6].

Sequencing depth significantly influences the accuracy of 16S rRNA sequencing in measuring microbial diversity. Greater sequencing depth can increase the observed diversity of a sample compared to lower depths. To address this issue, rarefaction is widely regarded as an effective strategy for controlling unequal sequencing depth when examining alpha- and beta-diversity, despite ongoing debates within the scientific community [7,8]. This method standardizes library size across samples by subsampling reads to a specified depth without replacement, thereby ensuring comparability. Samples with a total number of reads below the specified depth are excluded. This study performed rarefaction at a depth of 19,940 reads per sample, which was selected based on the minimum sequencing depth across all samples to ensure uniform sequencing coverage across all analyzed groups.

After rarefaction, alpha-diversity metrics (Chao1 and Shannon) and rarefaction curves were computed using QIIME 2- q2-diversity plugin. Furthermore, Chao1 and Shannon values were exported and analyzed utilizing IBM SPSS Statistics 27.0 (IBM software, Chicago, IL, USA). Two-way ANOVA was used for Chao1 and Shannon indexes. Rarefaction curves and bacterial relative abundances were visualized using R and RStudio with data exported from QIIME 2 [9,10].

The data underwent Hellinger transformation to standardize the data for ordination and Canonical Correspondence Analysis (CCA) was performed via ampvis2 R package [11,12]. CCA, introduced by ter Braak, revolutionized ordination methods by combining correspondence analysis (CA) with regression methodologies, enabling hypothesis testing in addition to exploratory analysis [13-16]. This advancement made CCA a powerful tool for understanding the influence of environmental factors on community composition, particularly in ecology and microbiome research [12,17].

Pairwise PERMANOVA (Permutational Multivariate Analysis of Variance) analysis, based on the Bray-Curtis dissimilarity index, was employed to identify significant differences in microbial community composition between the treatment groups [18]. PERMDISP (permutational analysis of multivariate dispersions) determines whether the distribution or spread of two sample groups differ significantly. A non-significant PERMDISP result, for instance, would show that the dispersions of the two sample groups are similar. This would give confidence to a significant PERMANOVA result because it is probably not the product of different group dispersions [19]. Both PERMANOVA and PERMDISP analyses were conducted using MicrobiomeAnalyst.

Based on the rarefied feature table, differential abundance testing was conducted using LEfSe (Linear Discriminant Analysis Effect Size) and the Kruskal-Wallis H-test. Linear Discriminant Analysis (LDA) score was calculated using the LEfSe algorithm by MicrobiomeAnalyst to identify taxa with significant differences in relative abundance across groups [20]. Taxa with an LDA score above threshold 2.0 and (P value < 0.05) were considered significantly enriched. The horizontal bars represent the effect size of each taxon, with longer bars indicating greater discriminatory power.

The Kruskal-Wallis H-test for the relative abundance comparison between subgroups was performed using Software for Analyzing Taxonomic or Metabolic Profiles (STAMP), with visualization including error bars to indicate standard deviations (SD) [21].

References:

1. Bolyen, E.; Rideout, J.R.; Dillon, M.R.; Bokulich, N.A.; Abnet, C.C.; Al-Ghalith, G.A.; Alexander, H.; Alm, E.J.; Arumugam, M.; Asnicar, F.; et al. Reproducible, interactive, scalable and extensible microbiome data science using QIIME 2. *Nat. Biotechnol.* **2019**, *37*, 852-857, doi:10.1038/s41587-019-0209-9.
2. Callahan, B.J.; McMurdie, P.J.; Rosen, M.J.; Han, A.W.; Johnson, A.J.; Holmes, S.P. DADA2: High-resolution sample inference from Illumina amplicon data. *Nat. Methods.* **2016**, *13*, 581-583, doi:10.1038/nmeth.3869.
3. Price, M.N.; Dehal, P.S.; Arkin, A.P. FastTree 2--approximately maximum-likelihood trees for large alignments. *PLoS One* **2010**, *5*, e9490, doi:10.1371/journal.pone.0009490.
4. Katoh, K.; Standley, D.M. MAFFT multiple sequence alignment software version 7: improvements in performance and usability. *Mol. Biol. Evol.* **2013**, *30*, 772-780, doi:10.1093/molbev/mst010.
5. Quast, C.; Pruesse, E.; Yilmaz, P.; Gerken, J.; Schweer, T.; Yarza, P.; Peplies, J.; Glockner, F.O. The SILVA ribosomal RNA gene database project: improved data processing and web-based tools. *Nucleic Acids Res.* **2013**, *41*, D590-596, doi:10.1093/nar/gks1219.
6. Bokulich, N.A.; Kaehler, B.D.; Rideout, J.R.; Dillon, M.; Bolyen, E.; Knight, R.; Huttley, G.A.; Gregory Caporaso, J. Optimizing taxonomic classification of marker-gene amplicon sequences with QIIME 2's q2-feature-classifier plugin. *Microbiome* **2018**, *6*, 90, doi:10.1186/s40168-018-0470-z.
7. McMurdie, P.J.; Holmes, S. Waste not, want not: why rarefying microbiome data is inadmissible. *PLoS Comput. Biol.* **2014**, *10*, e1003531.

8. Schloss, P.D. Waste not, want not: revisiting the analysis that called into question the practice of rarefaction. *Msphere.* **2024**, *9*, e00355-00323.
9. Team, R.S. RStudio: integrated development environment for R. *(No Title)* **2021**.
10. Team, R.C. RA language and environment for statistical computing, R Foundation for Statistical. *Computing.* **2020**.
11. Andersen, K.S.; Kirkegaard, R.H.; Karst, S.M.; Albertsen, M. ampvis2: an R package to analyse and visualise 16S rRNA amplicon data. *BioRxiv* **2018**, 299537.
12. Xia, Y.; Sun, J. Alpha Diversity. In *Bioinformatic and Statistical Analysis of Microbiome Data*, Xia, Y., Sun, J., Eds.; Springer International Publishing: Cham, 2023; pp. 289-333.
13. ter Braak, C.J.F. Canonical Correspondence Analysis: A New Eigenvector Technique for Multivariate Direct Gradient Analysis. *Ecology* **1986**, *67*, 1167-1179, doi:10.2307/1938672.
14. ter Braak, C.J.F. *Ordination*; 1995; Volume 5.
15. Gauch Jr, H.G.; Whittaker, R.H.; Singer, S.B. A comparative study of nonmetric ordinations. *J. Ecol.* **1981**, 135-152.
16. Gauch Jr, H.G. Noise reduction by eigenvector ordinations. *Ecology* **1982**, *63*, 1643-1649.
17. Wilmes, P.; Bond, P.L. The application of two-dimensional polyacrylamide gel electrophoresis and downstream analyses to a mixed community of prokaryotic microorganisms. *Environ. Microbiol.* **2004**, *6*, 911-920, doi:10.1111/j.1462-2920.2004.00687.x.
18. Bray, J.R.; Curtis, J.T. An ordination of the upland forest communities of southern Wisconsin. *Ecol. Monogr.* **1957**, *27*, 326-349.
19. Anderson, M.J.; Walsh, D.C. PERMANOVA, ANOSIM, and the Mantel test in the face of heterogeneous dispersions: what null hypothesis are you testing? *Ecol. Monogr.* **2013**, *83*, 557-574.
20. Chong, J.; Liu, P.; Zhou, G.; Xia, J. Using MicrobiomeAnalyst for comprehensive statistical, functional, and meta-analysis of microbiome data. *Nat. Protoc.* **2020**, *15*, 799-821, doi:10.1038/s41596-019-0264-1.
21. Parks, D.H.; Tyson, G.W.; Hugenholtz, P.; Beiko, R.G. STAMP: statistical analysis of taxonomic and functional profiles. *Bioinformatics* **2014**, *30*, 3123-3124, doi:10.1093/bioinformatics/btu494.
